# Axillary nodal metastasis and resection margins as predictors of Loco Regional Recurrence in Breast Cancer Patients

**DOI:** 10.4314/ahs.v22i1.15

**Published:** 2022-03

**Authors:** OO Ayandipo, OJ Adepoju, GO Ogun, OO Afuwape, OY Soneye, IB Ulasi

**Affiliations:** 1 Department of Surgery, College of Medicine, University of Ibadan/University College Hospital, Ibadan, Nigeria; 2 Department of Surgery, University College Hospital, Ibadan; 3 Department of Pathology, College of Medicine, University of Ibadan/ University College Hospital, Ibadan, Nigeria

**Keywords:** Recurrence, breast cancer, Ibadan, Axillary nodes, resection margins

## Abstract

**Background:**

Surgical resection margins (RM), axillary nodal involvement and lymph node ratio (LNR) determine loco-regional control (LRC) in breast cancer management. Late presentation precludes breast conservation therefore surgical option is usually mastectomy and adjuvant chemoradiation minimize loco-regional recurrence (LRR).

**Objective:**

We investigated the prognostic role of lymph nodes positive for malignancy (pN), LNR and RM on LRR of breast cancer in a tertiary hospital in Ibadan, Nigeria.

**Methods:**

Longitudinal cohort study of 225 females with breast carcinoma managed and followed up for 5-years with end point of LRR or not. Chi-square test and logistic regression analysis were used to evaluate the interaction of resection margin and proportion of metastatic lymph nodes with LRR. The receiver-operator curve was plotted to determine the proportion of metastatic lymph nodes which predicted LRR.

**Results:**

Ninety-nine percent had modified radical mastectomy and 163 (72.4%) had negative resection margins. A mean of 11 axillary lymph nodes were harvested at surgery. The age, positive resection margin and number of harvested nodes with malignant cells are associated with LRR. The overall 5-year LRR rate was 16%.

**Conclusion:**

LRR is dependent on lymph node involvement as well as and tumor aggressiveness.

## Introduction

Female breast cancer globally continues to witness increasing age-adjusted incidence rate^1^, as well as being a leading cause of disease-specific cancer mortality, even in high-income countries^2^,^3^. In Nigeria, breast cancer is the most common female malignancy and it accounts for the highest cancer related mortality^1^,^3^. The Ibadan cancer registry in 2012 reported similar findings in 2012 1. Social and cultural factors significantly influence health-seeking behaviour, attitude to orthodox treatment of breast cancer, uptake of breast cancer surgery - including breast-conserving surgery and mastectomy, adherence to adjuvant therapy as well as attrition from follow-up^3^,^4^,^5^. These are important determinants of recurrence and survivorship.

Breast cancer surgery is aimed at achieving microscopically free resection margins (RM), otherwise, adjuvant treatment with radio- and/or chemotherapy is imperative. In our environment, late presentation usually precludes breast conserving procedures with surgical choice tending more towards breast ablation and adjunct aggressive local disease control to minimize loco-regional recurrence (LRR). 6–10 In a series of 234 Korean women followed up after breast cancer surgery and adjuvant therapy, cases of non-pectoralis muscle/fascia positive RM were evaluated for re-excision. With a cumulative positive and ‘close’ (defined as tumor cells within 2mm) RM of 26.5%, the overall 5-year loco-regional disease control (LRC) rate was 88.8%, with recurrence occurring in axillary nodes, supra/infraclavicular region, internal mammary nodes and ipsilateral breast/chest wall, in descending order. 11 While surgical resection remains the pivot of LRC in the multidisciplinary approach to breast cancer care, axillary lymph nodal involvement remains a most crucial prognostic parameter,^11^,^12^ with a significantly higher recurrence risk found in breast cancer patients with >10 positive axillary nodes (pN).^10^, ^11^

An equally important prognostic index, lymph node ratio (LNR), defined as the proportion of retrieved lymph nodes positive for malignancy^13^,^14^ has a superior prognostic effect to total number of harvested nodes^12^ Furthermore, while authors have alluded to LNR>0.7 as an independent determinant of LRR, modification of staging models to include LNR has been advocated.^11^

This study investigates the prognostic role of pN, LNR and RM on LRR of breast cancer in a tertiary hospital in Ibadan, Nigeria.

## Methods

A cohort of 225 patients who underwent modified radical mastectomy or breast conserving surgery with axillary clearance for breast carcinoma at the Division of Surgical Oncology of the University College Hospital, Ibadan, between December 2009 and December 2014. Ethical approval was obtained from the Ministry of Health, Oyo State, Nigeria. Patients with established metastatic disease were excluded from the study because the aim of care in them is palliative and most of them would not be offered surgery. Treatment protocol was based on the European Society of Medical Oncology (ESMO) guideline 15.

Preoperative diagnosis was made from histology samples obtained by core needle biopsy and disease staging was done with plain chest radiography, abdominal ultrasonography and bone scintigraphy. Patients with American Joint Committee on Cancer (AJCC, 8th edition) stages I to III disease were recruited. Those with stage I and II breast cancer had quadrantectomy with axillary clearance while operable stage IIIA disease had mastectomy with axillary clearance. Patients with inoperable / locally advanced (stage III B and C) disease had neoadjuvant systemic +/- radiotherapy before mastectomy and axillary clearance.

The breast and axillary specimens were examined by breast pathologists, specifically for histological type and grade, axillary nodal metastasis and RM (defined as negative or positive). Immunohistochemistry was done to determine hormone receptor status. Adjuvant therapy comprised chemotherapy, radiotherapy, endocrine therapy, immunotherapy or a combination of these.

Following completion of treatment, the patients were followed up for a maximum period of five years. Outcomes of interest included local recurrence (defined by detection of cancer cells in the chest wall of the treated breast or axillary lymph nodes) and distant metastasis. This was assessed clinically and with the use of plain chest radiography, abdominal ultrasonography and bone scintigraphy. Any new breast cancer involving the ipsilateral breast is adjudged to be a recurrence. Statistical analysis was performed using Statistical Package for Social Sciences software (version 22; SPSS Inc. Chicago, IL). Descriptive statistics was used to examine the demographic and clinico-pathological profile of the patients. Chi-square test and logistic regression analysis were used to evaluate the interaction of age of participants, resection margin, number of harvested lymph nodes and proportion of metastatic lymph nodes with tumour recurrence. The receiver-operator (ROC) curve was plotted to determine the proportion of metastatic lymph nodes which predicted LRR.

Statistical significance was set at P <0.05

## Results

Two hundred and twenty-five (225) patients were recruited for the study. Table 1 shows their biodata. The age range of the patients was from 28 to 77 years with a median age of 47 years. The mean age was 48.6 ± 11.8 years.

**Table 1:** Bio-data

	Frequency (n = 225)	Percentage (%)
Age		
< 35 years	27	12.0
35 – 44 years	64	28.4
45 – 54 years	65	28.9
55 – 64 years	45	20.0
	24	10.7
21 – 30 years	3	1.3
31 – 40 years	64	28.4
41 – 50 years	69	30.4
51 – 60 years	44	19.6
61 – 70 years	36	16.0
71 – 80 years	9	4.0

### Clinico-pathologic profile

The average tumour size was 6 cm, with two-thirds of patients having tumour sizes above 5cm (table 2). Nine of every ten participants had invasive carcinoma, not otherwise specified. The commonest tumor grade was the intermediate variety (49.8%; n = 112), followed by lowgrade breast cancers. Almost all of the participants had modified radical mastectomy (99%; n= 222).

**Table 2:** Clinico-pathologic profile

	Frequency (n = 225)	Percentage (%)
Breast Cancer Laterality		
Right	104	46.2
Left	121	53.8
Tumour Size (cm (0cm)	3	1.3
cm)	12	5.3
cm)	63	28.0
cm)	147	65.3
Histological Type		
Invasive carcinoma NOS	203	90.2
Invasive lobular carcinoma	13	5.8
Phyllodes tumour	3	1.3
DCIS	6	2.7
Tumour Grade		
Low grade	39	17.3
Intermediate grade	112	49.8
High grade	36	16.0
Not stated	38	16.9
Resection Margin		
Free	163	72.4
Involved	60	26.7
Unknown	2	0.9
Immuno-histochemistry		
Luminal A	69	30.7
Luminal B	6	2.7
Her-2-enriched	21	9.3
Triple Negative	35	15.6
Not stated	94	41.7
Nature of Surgery		
MRM	222	98.7
Quadrantectomy + Axillary Clearance	3	1.3

NOS ;not otherwise specified: MRM: modified radical mastectomy

The resection margin was free in 72% of patients with the most common immunohistochemical type being Lumina A, followed by the triple negative variety. Immunohistochemical type and tumour size was not significantly associated with resection margin (p = 0.514 and p = 0.074 respectively)

Majority of patients (80.9%; n=182) had between 4 – 11 axillary lymph nodes harvested with a mean of 11 nodes (Table 3).

**Table 3:** Axillary lymph nodal status, neo-adjuvant treatment and outcomes

	Frequency (n = 225)	Percentage (%)
Harvested Nodes		
0	12	5.3
1 – 3	31	13.8
4 – 9	72	32.0
	110	48.9
Metastatic Nodes		
0	103	45.8
pN1: 1 – 3	47	20.9
pN2: 4 – 9	63	28.0
pN3:	12	5.3
Neo-Adjuvant Therapy		
None	107	47.6
Chemotherapy	115	51.1
Chemotherapy + Radiotherapy	3	1.3
Adjuvant Therapy		
None	14	6.2
Chemotherapy	117	52.0
Chemotherapy + Radiotherapy	78	34.7
Radiotherapy alone	16	7.1
Both neo-adjuvant and adjuvant therapy	106	47.1
Evidence of Recurrence		
Yes	36	16.0
No	183	81.3
Unknown	6	2.7
Survival status		
Alive	147	65.3
Dead	63	28.0
Unknown	15	6.7

Nearly half of the patients (51.1%; n= 115) had neoadjuvant chemotherapy alone whereas as high as 47.6% had no neoadjuvant care.

### Treatment outcomes and prognostic factors

Although there was no cancer recurrence in 81.3% of patients, as high as 28% mortality was recorded as shown in table 3. Out of this, 10.7% had both recurrence with mortality while while the remaining 11.3% had just mortality with no recurrence

Factors significantly associated with recurrence of cancer include age of the participants (p = 0.019), resection margin (p = 0.001), number of harvested nodes (p = 0.002) and number of harvested nodes with malignant cells (p < 0.01) (Table 4)

**Table 4:** Factors associated with recurrence

	No recurrence	Recurrence	Chi-square (p-value)	Logistic Regression OR (95% CI)	p-value
Age					
< 35 years	24 (88.9%)	3 (11.1%)	11.73 (0.019)	1	
35 – 44 years	49 (80.3%)	12 (19.7%)		1.96 (0.51 – 7.60)	0.331
45 – 54 years	56 (86.2%)	9 (13.8%)		1.29 (0.32 – 5.17)	0.723
55 – 64 years	39 (92.9%)	3 (7.1%)		0.62 (0.12 – 3.30)	0.571
> 65 years	15 (62.5%)	9 (37.5%)		4.80 (1.12 – 20.61)	0.035
Resection Margin					
Free	139 (88.5%)	18 (11.5%)	10.78 (0.001)	1	
Involved	42 (70%)	18 (30%)		3.31 (1.58 – 6.93)	0.01
Harvested Nodes					
0	9 (75%)	3 (25%)	14.75 (0.002)	1	
1 – 3	28 (90.3%)	3 (9.7%)		0.32 (0.06 – 1.88)	0.321
4 – 9	33 (47.8%)	36 (52.2%)		0.19 (0.04 – 0.91)	0.037
	77 (76.2%)	24 (23.8%)		1.24 (0.30 – 5.02)	0.768
Metastatic Nodes					
0	88 (90.7%)	9 (9.3%)	38.60 (< 0.01)	1	
1 – 3	35 (74.5%)	12 (25.5%)		2.67 (0.97 – 7.30)	0.056
4 – 9	57 (90.5%)	6 (9.5%)		1.03 (0.35 – 3.05)	0.96
	3 (25%)	9 (75%)		29.33 (6.71 – 128.31)	< 0.001
Metastatic Nodes					
No positive node	88 (90.7%)	9 (9.3%)	6.50 (0.011)	1	
1 or more positive node(s)	95 (77.9%)	27 (22.1%)		1.15 (1.05 – 1.26)	0.002

Patients aged 65 years or more were 5 times more likely to have cancer recurrence compared to those less than 35 years (OR = 4.8, p = 0.035) while those with a positive RM were thrice more likely to have cancer recurrence than those with a free resection margin (OR = 3.31, p = 0.01). The presence of 10 or more harvested nodes increases the odds of recurrence 1.2 times compared to those with no harvested lymph node (OR = 1.24, p = 0.768).

The presence of 1 or more positive lymph node increases by 1.2 times the likelihood cancer recurrence than those with no positive node (OR = 1.15, p = 0.002). Only 9% of those with no positive node had cancer recurrence.

**Fig 1:**
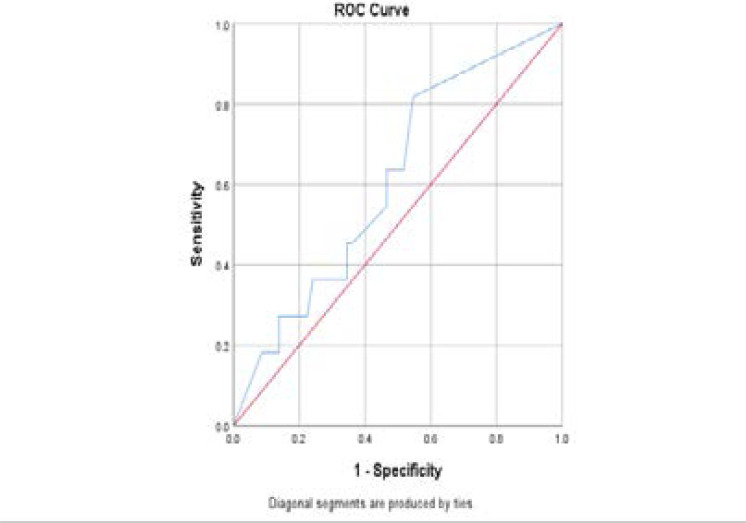
ROC curve for predicting recurrence using proportion of metastatic lymph nodes

The proportion of metastatic lymph nodes was 33% in patients with recurrent cancer while only 14% of metastatic lymph nodes were recorded in those with no evidence of cancer recurrence (p = 0.038). With a 56% sensitivity and specificity of 52%, the cut-off point of the proportion of metastatic lymph node that predicts cancer recurrence was 30% (Fig 1).

Patients with 1 – 3 metastatic nodes had 61% hazard rate from breast cancer (HR = 0.61, p = 0.235). This risk is 3.6 times higher in those with 4 – 9 metastatic nodes (HR = 2.19, p = 0.013), and 52 times higher in those with 10 or more metastatic nodes (HR = 31.62, p < 0.001). The above risk becomes higher when the hazard rate is adjusted. It was also noted that tumour size was not associated with tumour recurrence statistically (p = 0.894)

## Discussion

In terms of age distribution, tumor sidedness and tumor size, the pathologic summary of our series is in keeping with findings in literature. 6, 9, 16–18 Histologic subtypes of breast cancer in our cohort (90% being invasive ductal carcinoma) tallies with findings in Korea and Brazil; 88.5%11 and 87%^12^ respectivelyand also locally, while tumor grade distribution – two-thirds being intermediate/high grade, simulates the pattern obtained from other studies in the sub-region.^17^–^20^

Also, we did not identify a consistent pattern of breast cancer laterality in previous studies done in Nigeria.^17^

Even though almost three-quarters (72.4%) of our surgical specimens have histologically tumor-free margins - a value comparable with other series done in a similar clime (80.4%) and a developed clime (74%)^6^,^21^, we did not perform a routine re-operation for microscopic residual cancer found in 60 patients (26.7%). Rather, adjuvant chemoradiation therapy was considered adequate for residual microscopic tumor based on the treatment protocol. However, ipsilateral breast/post-mastectomy breast bed irradiation was not taken as a substitute to optimal oncologic resection, but as an adjunct to reduce the risk of ipsilateral breast tumor recurrence(IBTR).^22^–^24^ While over one-thirds of the study population had tumors <5cm (T0-2 tumors), only a mere 1.3% (3 patients) had breast conserving surgery. This is as a result of surgeon and patient's preference for ablative procedure based on the ESMO treatment guideline 15 as well as the presence of large tumor-breast ratio and precarious access to adjuvant radiation therapy due to relative unavailability, logistic factors and almost inevitably, cost in our environment. Since the risk of positive RM is known to increase with higher tumor grade and category,^9^ it is therefore striking that our cohort, with majority of patients (65.3%) with T3-4 tumors had a comparable positive RM with another series in which 22.2% were T3-4 (72.4% vs 73.5% respectively).^11^ However, the fact that 27.8% of the 234 patients in the series by Kim et al had breast-conserving surgeries keeps the pattern in perspective, especially because conservative breast resections presents a significantly higher positive RM and IBTR.^6^,^9^ Furthermore, positive RM is associated with lower 10-year disease-free survival and overall survival rates than mastectomy.^6^

In this study, report is made of margin status of the final therapeutic procedure performed in cases of sequential surgeries. For instance, documentation of quadrantectomy margins is made for patients who had a prior excision of malignant breast lump following an inconclusive pre-operative histology of core needle biopsy specimens. The argument on whether ipsilateral breast tumor recurrence from positive post-resection margin directly causes systemic recurrence remains largely unresolved,6 especially because many breast oncologists submit that breast cancer is a systemic disease from the outset,^25^ and that LRR is an indicator of adequate local therapy and tumor aggressiveness. Therefore, the mechanism by which positive RM indicates worse outcomes following breast cancer surgery may be from secondary tumor dissemination from local recurrence or local recurrence as a predictor of unfavorable tumor biology ab initio or a combination of these factors.

Following breast-conserving surgery, IBTR rates have been found to differ widely between oncology centers.^26^ The importance of completeness of scheduled multimodal treatment plan, as well as availability of a full complement of skilled multidisciplinary team personnel is therefore crucial to achieving optimal results following breast cancer treatment.

In this study, the overall median five-year survival was 65.3%, as compared with 79% reported in some HICs^27^. Despite instituting multimodal approach to breast cancer care, outcome figures in our cohort may not compare fairly with those from HICs due to unfavourable tumor grade and immunohistochemical pattern in a high proportion of premenopausal patients,^8^,^17^ as well as failure to complete recommended adjuvant treatment plan as a result of logistic challenges and unaffordable cost implications^18^. It is important to note that Ghana, sub-saharan Africa country reported a five-year survival rate of 84.7% comparable with most high income countries in a retrospective study done during the same period in patients with breast cancer. This may be due to the fact that all the patients recruited completed their proposed care before being followed-up to assess outcome^21^.

In this study, 48.9% had >10 axillary nodes harvested, overall mean being 11 nodes, less than 15 (2–31) reported by Abass et al in Sudan, 23 19 (6–77) found by Tonellotto12 and 26 (10–61) documented in South Korean women. 11 The lower lymph node yield in our series may be attributable to the fact that more than 50% of our patients had neoadjuvant chemotherapy which has been shown to reduce the number of lymph nodes retrieved in axillary dissection specimen^28^

It has been reported that axillary lymph node dissection is adjudged efficacious when >10 nodes are retrieved, 11,29,30 an assertion also further strengthened by German S3 guidelines^31^ despite awaiting further credence by high-quality evidence.^32^ Furthermore, evidence continues to accumulate that lymph node ratio is a more reliable predictor of loco-regional recurrence and survival than absolute number of harvested lymph nodes. In this study, 48.9% had >10 axillary nodes harvested, overall mean being 11 nodes, less than 15 (2–31) reported by Abass et al in Sudan, 21 19 (6–77) found by Tonellotto^12^ and 26 (10–61) documented in South Korean women.^11^

Ebner et al reported that the 12.1% of 2992 patients who had <10 nodes retrieved in their study following ALND were older, postmenopausal, had higher proportion of Luminal A tumors with lower hormone receptor positivity and lower tumor grade.^32^ They therefore submitted that the lymph node count is probably arbitrary as there was no significant difference in the survival and recurrence outcomes of breast cancer patients with <10 and >10 retrieved axillary nodes.

After adjusting for confounders in a multivariate logistic regression analysis, age of the participants, resection margin and number of harvested nodes with malignant cells were factors significantly associated with recurrence of cancer. While we found that patients aged 65 years or more 5 times more likely to have cancer recurrence compared to those younger than 35 years (OR = 4.8, p = 0.035), this is at variance with findings in literature in which younger premenopausal patients who tend to opt for breast conserving procedures despite relatively unfavorable tumor biology develop more recurrence.7 Factors responsible for this could include failure to follow through adjuvant therapy by the older patients. Other factors associated with recurrence mirror the pattern obtained in literature.^7^,^12^

The trade-off plot of the receiver operator characteristics (ROC) present classifiers with a sensitivity and specificity of 56% and 52% respectively, the cut-off proportion of histologically positive lymph node predicting disease-specific 5-year recurrence being 30%.

## Conclusion

Despite placing priority on adequate oncologic resection coupled with adjunct treatment in a cohort of breast cancer patients with pathologic summary comparable to those in other Oncology centres, we found that increasing age of the participants, increase number of metastatic harvested nodes, and positive RM are associated with LRR. The lower lymph nodal yield can be attributed to good uptake of neoadjuvant chemotherapy.

## Limitations

Challenges in delivery of multimodal adjuvant treatment regimen (especially radiotherapy) to patients due to unavailability and lack of funds for care by patients. Failure of completion of proposed adjuvant care by some elderly patients might have contributed to the increased recurrence rate seen in them in this study
